# Apigenin Ameliorates Scopolamine-Induced Cognitive Dysfunction and Neuronal Damage in Mice

**DOI:** 10.3390/molecules26175192

**Published:** 2021-08-27

**Authors:** Yeojin Kim, Jihyun Kim, Meitong He, Ahyoung Lee, Eunju Cho

**Affiliations:** 1 Department of Food Science and Nutrition & Kimchi Research Institute, Pusan National University, Busan 46241, Korea; kimyj1231@naver.com (Y.K.); llissunll@gmail.com (J.K.); skyham16@gmail.com (M.H.); 2 Neural Circuit Research Group, Korea Brain Research Institute, Daegu 41062, Korea; 3 Department of Food Science, Gyeongsang National University, Jinju 52725, Korea

**Keywords:** apigenin, cognitive ability, oxidative stress, neuronal damage, scopolamine

## Abstract

We investigated the protective effect and mechanisms of apigenin against cognitive impairments in a scopolamine-injected mouse model. Our results showed that intraperitoneal (i.p.) injection of scopolamine leads to learning and memory dysfunction, whereas the administration of apigenin (synthetic compound, 100 and 200 mg/kg/day) improved cognitive ability, which was confirmed by behavioral tests such as the T-maze test, novel objective recognition test, and Morris water maze test in mice. In addition, scopolamine-induced lipid peroxidation in the brain was attenuated by administration of apigenin. To further evaluate the protective mechanisms of apigenin on cognitive and memory function, Western blot analysis was carried out. Administration of apigenin decreased the B-cell lymphoma 2-associated X/B-cell lymphoma 2 (Bax/Bcl-2) ratio and suppressed caspase-3 and poly ADP ribose polymerase cleavage. Furthermore, apigenin down-regulated the β-site amyloid precursor protein-cleaving enzyme, along with presenilin 1 (PS1) and PS2 protein levels. Apigenin-administered mice showed lower protein levels of a receptor for advanced glycation end-products, whereas insulin-degrading enzyme, brain-derived neurotrophic factor (BDNF), and tropomyosin receptor kinase B (TrkB) expression were promoted by treatment with apigenin. Therefore, this study demonstrated that apigenin is an active substance that can improve cognitive and memory functions by regulating apoptosis, amyloidogenesis, and BDNF/TrkB signaling pathways.

## 1. Introduction

The brain, a central organ of the human body, is responsible for memory and cognitive function [1]. Behavioral abnormalities and cognitive decline in the central nervous system (CNS) are linked to oxidative stress [2]. This means that the overproduction of reactive oxygen species (ROS) leads to neuron damage in the hippocampus, causing the impairment of cognitive and memory functions [3]. Moreover, memory loss and cognitive impairment are associated with neurological problems in brain function related to neurodegenerative diseases such as Alzheimer’s disease (AD) [4]. Mainly, the pathogenesis of AD includes amyloid beta (Aβ) production, phosphorylated tau protein, the accumulation of nerve fiber tangles, and oxidative stress [5,6]. Among them, the main characteristic of AD is the accumulation of Aβ plaque and phosphorylated tau protein in neurons of the brain. Aβ plaque is produced from amyloid precursor protein (APP), which is sequentially cleaved by β-secretase (BACE) and γ-secretase (presenilin 1 and 2) [7]. The principal component of senile plaques is Aβ peptides that act as neurotoxins and interfere with synaptic communication [8]. Furthermore, the toxicity of Aβ is related to increased oxidative stress and lipid peroxidation in neurons [9]. Oxidative stress caused by overproduction of ROS and reactive nitrogen species (RNS), such as superoxide anion radical (O_2_^–^), hydroxyl radical (∙OH), nitric oxide (NO), and peroxynitrite (ONOO^–^), can cause oxidation of lipids, proteins, cell membranes, and DNA, eventually leading to cellular aging and deformation [10,11]. In particular, the brain requires more oxygen to perform hippocampal synaptic functions, though the brain is vulnerable to oxidative stress [12]. Hence, the overproduction of ROS contributes to AD progression, which causes oxidative damage by a mechanism of neurotoxicity and induces memory deficit [13].

Scopolamine is a widely used in vivo model for inducing damage to the nerve system, resulting in memory loss and cognitive impairment mimicking those observed in AD [14,15]. According to previous studies, injection of scopolamine induced oxidative stress, along with memory and cognitive deficits, in the hippocampus of a Wistar rat and Institute of Cancer Research (ICR) mouse model [16,17,18,19]. Furthermore, a recent study confirmed that scopolamine-injected mice showed a decrease in brain-derived neurotrophic (BDNF)/tropomyosin receptor kinase B (TrkB) expressions, resulting in long-term and working memory deficits [20]. Therefore, we used the scopolamine-injected mouse model to assess potential agents for the treatment of AD.

The current drugs clearly cannot treat the progression of AD, but there are some drugs (donepezil, rivastigmine, and galantamine) used for temporary delay of AD progress [21]. However, these drugs have been reported to show side effects such as vomiting, loss of appetite, and insomnia [22]. Therefore, researchers have focused on examining the prevention of AD with natural products with beneficial effects deriving from their daily consumption [20]. Apigenin (4′,5,7-trihydroxyflavone), a less-toxic and non-mutagenic compound, is an edible, plant-derived flavonoid abundant in various vegetables and fruits, such as parsley, celery, onions, and oranges. According to the USDA (United States Department of Agriculture) database, parsley has a significant amount of apigenin [23,24]. Apigenin has been reported to exhibit anti-inflammation [25], anti-apoptosis [26], and free radical-scavenging activities [27] in vitro and in vivo. Previous study [28] indicated that oral administration of apigenin protects from Aβ_25–35_–induced oxidative damage of the neurovascular system by regulation of BDNF/TrkB levels in amnesic mice. In addition, apigenin reduced Aβ deposition in the APP/PS1 transgenic AD mouse model [29]. Despite the therapeutic effect of apigenin on neuronal function, the protective role of apigenin against scopolamine-induced cognitive impairment and potential mechanisms has not yet been studied.

In the present study, we investigated the protective effect of apigenin, which was obtained from Sigma-Aldrich Co., Ltd. (St Louis, MO, USA), against memory and cognitive deficits via behavioral tests (T-maze, novel objective recognition, and Morris water maze tests) in mice injected with scopolamine. In addition, we studied whether apigenin attenuates oxidative stress by measuring the malondialdehyde levels, and protective mechanisms of apigenin on the apoptosis, amyloidogenic, and BDNF/TrkB pathways were also analyzed in the brains of mice.

## 2. Results and Discussion

### 2.1. Effect of Apigenin on the Body Weight Change and Organs Weight in Scopolamine-Injected Mice

Decline in the body weight and internal organ weight is a simple and sensitive index with which to determine toxicity after injection of scopolamine or treatment of samples. The changes in body weight and organ weights were measured in scopolamine-injected mice (Table 1). Before oral administration of apigenin, the initial body weights among the five groups did not show significant differences, and we found no statistical differences in the body weight gain for each group during the experiment. After dissection, there were no significant differences in organ (brain, kidney, and liver) weights among all groups, indicating that mice did not have any problems, such as inflammation or edema. Moreover, these results demonstrated that scopolamine, apigenin, and donepezil pose no detectable abnormalities to the brain, liver, and kidney.

### 2.2. Effect of Apigenin on the T-Maze Test in Scopolamine-Injected Mice

**Figure 1:**
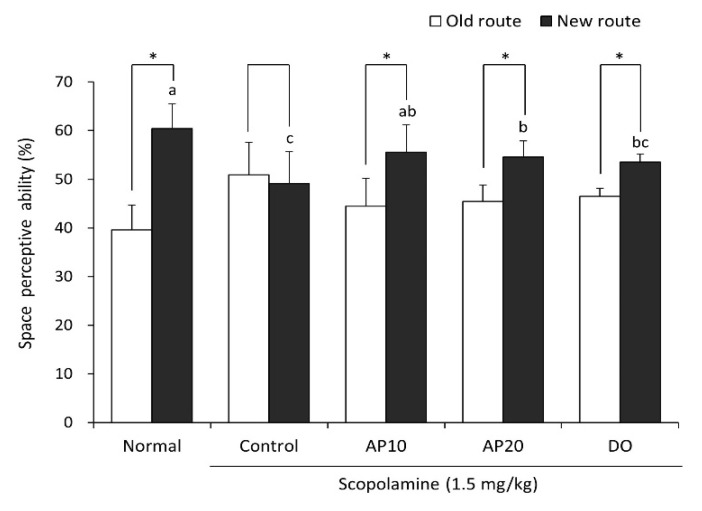
Effect of apigenin on spatial alternation in the T-maze test. Values are the mean ± SD. ^a^^–c^ The different letters among groups represent significant differences (*p* < 0.05) by Duncan’s multiple range test. * The space perceptive abilities for old and new routes are significantly different as determined by Student’s *t*-test (*p* < 0.05). Normal: oral administration of drinking water + 0.9% NaCl i.p.; Control: oral administration of drinking water + scopolamine 1.5 mg/kg i.p.; AP10: oral administration of apigenin 10 mg/kg + scopolamine 1.5 mg/kg i.p.; AP20: oral administration of apigenin 20 mg/kg + scopolamine 1.5 mg/kg i.p.; and DO: oral administration of donepezil 5 mg/kg + scopolamine 1.5 mg/kg i.p.

The T-maze test was performed to evaluate the spatial memory function of mice. Exploring a new route rather than a familiar route is considered to demonstrate improved space perception ability [30]. As presented in Figure 1, the scopolamine-injected control group did not show a significant difference in spatial exploration for the old route and new route. In contrast, the non-scopolamine treated normal group demonstrated significantly increased exploration in the new route (60.4%) compared with the old route (39.6%), suggesting that injection of scopolamine leads to impairment of cognition and a memory deficit in mice. The AP10 and AP20 groups significantly increased the new route exploration from 44.5% to 55.5% and from 45.5% to 54.6%, respectively. The DO group showed an increase in exploration between the old route and new route from 46.5% to 53.5%. These results showed that apigenin-administered mice improved spatial cognitive ability against scopolamine.

### 2.3. Effect of Apigenin on Novel Object Recognition Test in Scopolamine-Injected Mice

**Figure 2:**
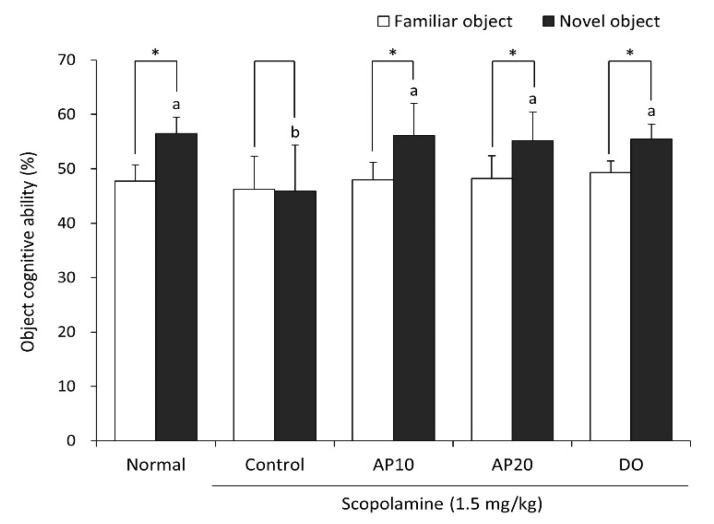
Effect of apigenin on recognition memory in the novel object recognition test. ^a–b^ The different letters among groups represent significant differences (*p* < 0.05) by Duncan’s multiple range test. * The space perceptive abilities for old and new routes are significantly different as determined by Student’s *t*-test (*p* < 0.05). Normal: oral administration of drinking water + 0.9% NaCl i.p.; Control: oral administration of drinking water + scopolamine 1.5 mg/kg i.p.; AP10: oral administration of apigenin 10 mg/kg + scopolamine 1.5 mg/kg i.p.; AP20: oral administration of apigenin 20 mg/kg + scopolamine 1.5 mg/kg i.p.; and DO: oral administration of donepezil 5 mg/kg + scopolamine 1.5 mg/kg i.p.

A novel object recognition test was conducted to confirm the memory retention, based on evidence that rodents prefer to explore novel objects rather than familiar objects [31]. To elucidate the effect of apigenin on object recognition ability, a novel object recognition test was carried out (Figure 2). The scopolamine-treated control group showed no significance in exploration between familiar and novel objects, indicating that scopolamine induced the impairment of their object recognition ability. However, the untreated-normal group significantly increased the number of touches for new object (56.5%) compared to old object (47.7%). Moreover, AP10 and AP20 groups significantly increased in their exploration of the novel object, from 47.9% to 56.2% and from 48.2% to 55.2%, respectively. These results suggest that the administration of apigenin improved scopolamine-induced object recognition deficit.

### 2.4. Effect of Apigenin on Morris Water Maze Test in Scopolamine-Injected Mice

**Figure 3:**
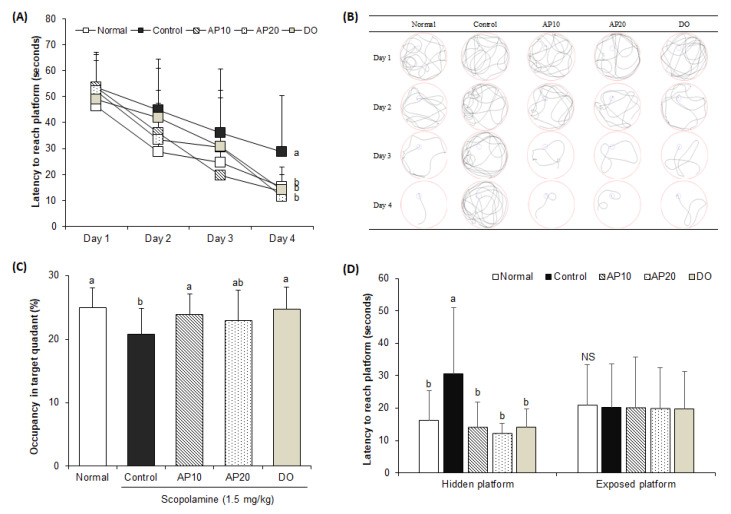
Effects of apigenin on latency to reach the hidden platform during four days in the Morris water maze test (**A**), representative swimming-tracking paths of the Morris water maze test (**B**), occupancy time to stay in target quadrant on the final day in the Morris water maze test (**C**), latency to reach the hidden and exposed platforms on the final day in the Morris water maze test (**D**). Values are the mean ± SD. ^a^^–b^ The different letters among groups represent significant differences (*p* < 0.05) by Duncan’s multiple range test. NS: non-significance. Normal: oral administration of drinking water + 0.9% NaCl i.p.; Control: oral administration of drinking water + scopolamine 1.5 mg/kg i.p.; AP10: oral administration of apigenin 10 mg/kg + scopolamine 1.5 mg/kg i.p.; AP20: oral administration of apigenin 20 mg/kg + scopolamine 1.5 mg/kg i.p.; and DO: oral administration of donepezil 5 mg/kg + scopolamine 1.5 mg/kg i.p.

The Morris water maze test was conducted to analyze the effect of apigenin on long-term and spatial memory ability in mice [32]. A swimming-tracking path was measured for four days using a SMART video tracking program (Figure 3A,B). On the first day, the swimming-tracking paths of all mice showed a long-complicated route, as all groups were untrained. As the experiment of the learn-training session progressed, however, the normal group showed a shorter swim trace to reach the hidden platform during the four days. In contrast, the scopolamine-injected control mice indicated no significant differences in the length and shape of the swim trace for the Morris water maze test during the four days. However, the AP10, AP20, and DO groups revealed progressively shorter swim traces, indicating that treatments of apigenin and donepezil effectively protected spatial learning and memory function against scopolamine in mice. In addition, these results suggested that the longer swim trace led to a longer latency to search for the hidden platform in the scopolamine-injected control group compared with the untreated normal group during the training days. However, the normal group showed that the latency to find the hidden platform was gradually shortened compared with the control group over four days. In addition, the AP10, AP20, and DO groups significantly decreased the latency to reach the hidden platform, compared with the control group. On the last day of the experiment, the spent time in the target quadrant with a visual sign was recorded after the elimination of the hidden platform (Figure 3C). The control group spent the shortest time in the target quadrant (20.8%) compared with the normal group (25.0%). In contrast, AP10, AP20, and the donepezil-administered group spent a significantly longer time in the target quadrant, showing 23.9%, 23.0%, and 24.7%, respectively. As shown in Figure 3D, the time to reach the exposed platform was not significantly different among all the groups, suggesting that the effect of apigenin on cognitive improvement was not related to visual or swimming abilities, but learning and memory abilities. Similarly, previous studies also demonstrated that apigenin improved spatial working memory function in behavioral tasks such as the Y-maze and Morris water maze tests in Aβ_25__–35_- or isoflurane-induced in vivo model [33,34]. These findings demonstrated that apigenin could ameliorate cognitive dysfunction induced by scopolamine.

### 2.5. Effect of Apigenin on Lipid Peroxidation in the Brain of Scopolamine-Injected Mice

**Figure 4:**
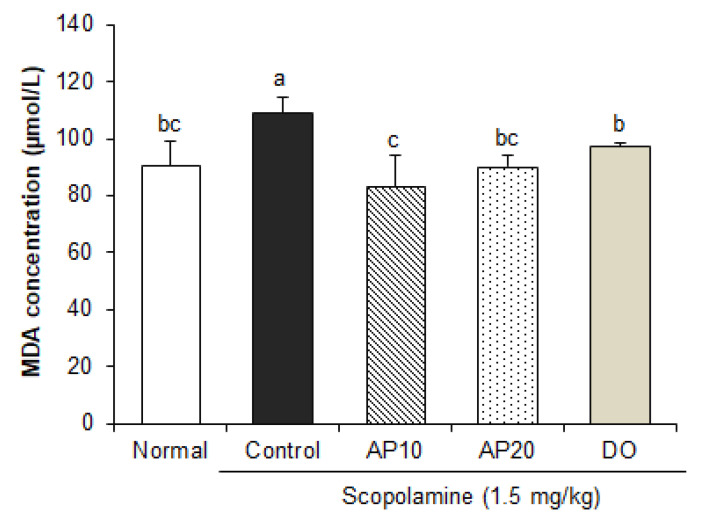
Effect of apigenin on levels of MDA induced by scopolamine in the brain of mice. Values are the mean ± SD. ^a–^^c^ The different letters among groups represent significant differences (*p* < 0.05) by Duncan’s multiple range test. NS: non-significance. Normal: oral administration of drinking water + 0.9% NaCl i.p.; Control: oral administration of drinking water + scopolamine 1.5 mg/kg i.p.; AP10: oral administration of apigenin 10 mg/kg + scopolamine 1.5 mg/kg i.p.; AP20: oral administration of apigenin 20 mg/kg + scopolamine 1.5 mg/kg i.p.; and DO: oral administration of donepezil 5 mg/kg + scopolamine 1.5 mg/kg i.p.

It has been well established that oxidative stress is implicated in the progression of AD [35]. Abnormal proteolytic processing of APP and secretase enzymes is involved in Aβ deposition, leading to oxidative stress, neuronal cell death, and cognitive impairment. Aβ-mediated oxidative stress can cause metabolic alteration in the brain, such as lipid peroxidation and ROS production [36]. Previous studies have shown that injection of scopolamine caused oxidative stress by decreasing antioxidant enzymes’ activity (superoxide dismutase or glutathione peroxidase) and increasing lipid peroxidation in the brain [37,38]. Malondialdehyde (MDA), an end-product of lipid peroxidation, is considered to be one of the markers of radical generation. To investigate whether apigenin protects scopolamine-induced oxidative stress, we measured the levels of MDA in the brains of mice. As shown in Figure 4, the mice injected with scopolamine showed an increase in the MDA contents when compared to non-injected mice. On the other hand, supplementation of apigenin (10 and 20 mg/kg) showed a significant decrease in MDA levels, compared to the control group. These findings are consistent with previous studies showing that treatment of apigenin provides protection against oxidative stress through reduction in MDA concentrations in a diabetes-associated cognitive decline mouse model [39]. In addition, the ROS and MDA levels were significantly decreased by apigenin, resulting in the protection of neuronal apoptosis [40]. Therefore, our results suggest that apigenin may contribute to the improvement of scopolamine-induced learning and memory impairment by attenuating oxidative stress.

### 2.6. Effect of Apigenin on Apoptosis-Related Protein Expressions in the Brains of Scopolamine-Injected Mice

Oxidative stress induced by scopolamine can cause inflammation and apoptosis in the brain, leading to the death of hippocampal neurons [41,42]. The B-cell lymphoma (Bcl)-family is known as an important mediator of apoptosis in the nervous system [43]. The Bcl-2 protein acts as anti-apoptosis factor via suppression of cytochrome C release in mitochondria, whereas the Bcl-2-associated X (Bax) protein is related to induction of apoptosis [44]. These proteins regulate cytochrome C release or mitochondrial membrane depolarization, thereby deciding cell death [45]. Moreover, cytochrome C can cleave and activate caspase-3, which directly degrades poly ADP ribose polymerase (PARP), a key protein factor for apoptosis [46,47,48]. Therefore, caspase-3 and PARP are being studied as the most important executors of apoptosis. A previous study showed that scopolamine increased levels of Bax and caspase-3, and reduced levels of Bcl-2 proteins in the brains of mice [49]. In addition, scopolamine increased expression of PARP and caspase-3 in the brain tissue of the dementia animal model, resulting in neuronal apoptosis [50].

**Figure 5:**
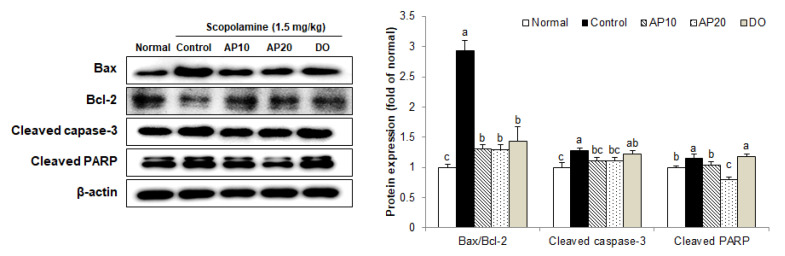
Effect of apigenin on levels of Bax/Bcl-2, cleaved caspase-3, and PARP protein expression induced by scopolamine in the brain of mice. ^a–^^c^ The different letters among groups represent significant differences (*p* < 0.05) by Duncan’s multiple range test. Results of expression were calculated using β-actin as the standard. Normal: oral administration of drinking water + 0.9% NaCl i.p.; Control: oral administration of drinking water + scopolamine 1.5 mg/kg i.p.; AP10: oral administration of apigenin 10 mg/kg + scopolamine 1.5 mg/kg i.p.; AP20: oral administration of apigenin 20 mg/kg + scopolamine 1.5 mg/kg i.p.; and DO: oral administration of donepezil 5 mg/kg + scopolamine 1.5 mg/kg i.p.

To confirm the protective mechanisms of apigenin against scopolamine-induced neuronal damage, the levels of apoptosis-related protein expression were analyzed in a mouse brain (Figure 5). The ratio of Bax/Bcl-2 was significantly increased in the control group treated with scopolamine, compared with the normal group. However, supplementation with apigenin significantly decreased the Bax/Bcl-2 expression levels. In addition, the levels of cleaved caspase-3 and cleaved PARP protein expression were up-regulated in the control group, compared with the scopolamine-untreated normal group. In contrast, the AP10 and AP20 groups showed lower levels of these apoptosis-related protein expressions. Several studies also reported the protective activity of apigenin from apoptosis. Apigenin reduced caspase-3 and Bax levels in apoptotic neurons in early brain injury after subarachnoid hemorrhage [40]. Moreover, treatment of apigenin suppressed neuronal cell death by inactivation of caspases (-8, -9, and -3) and cleavage of PARP [51]. Thus, these results indicate that apigenin had a protective effect on neuronal damage through inhibition of the apoptosis signaling pathway in AD mouse models induced by scopolamine.

### 2.7. Effect of Apigenin on Amyloidogenic Pathway in the Brains of Scopolamine-Injected Mice

One of the key pathological causes of AD is the overproduction of Aβ, which induces apoptosis and oxidative stress, leading to neuronal damage and cognitive impairment [52]. Although mechanisms of Aβ-induced damage are unclear, it is related to abnormal enzyme cascades in which Aβ forms neurotoxic species and leads to AD progression [53,54]. Aβ is produced by the degradation of APP through γ-secretase and BACE [55]. The first step of APP cleavage occurs by BACE, and then generates C-terminal fragment (CTF) intermediates [56]. Subsequently, the endopeptidase γ-enzyme cleaves the CTF to produce Aβ and other metabolites [57]. Therefore, expression levels of BACE and γ-secretase are expected to play an important role in producing Aβ.

**Figure 6:**
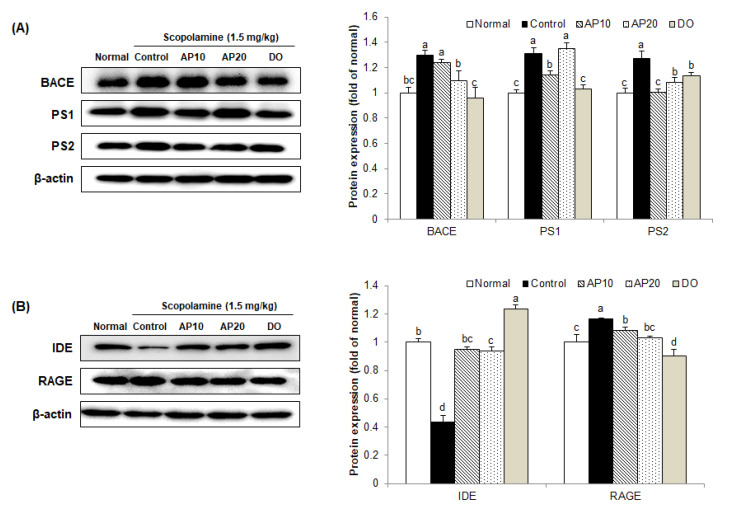
Effect of apigenin on levels of BACE, PS1, and PS2 (**A**), IDE and RAGE (**B**) protein expression induced by scopolamine in the brain of mice. Values are the mean ± SD. ^a–^^d^ The different letters among groups represent significant differences (*p* < 0.05) by Duncan′s multiple range test. Results of expression were calculated using β-actin as the standard. Normal: oral administration of drinking water + 0.9% NaCl i.p.; Control: oral administration of drinking water + scopolamine 1.5 mg/kg i.p.; AP10: oral administration of apigenin 10 mg/kg + scopolamine 1.5 mg/kg i.p.; AP20: oral administration of apigenin 20 mg/kg + scopolamine 1.5 mg/kg i.p.; and DO: oral administration of donepezil 5 mg/kg + scopolamine 1.5 mg/kg i.p.

To elucidate the effect of apigenin on the amyloidogenic pathway, Aβ generation-related protein expression was evaluated in a scopolamine-injected mouse brain. Figure 6 showed that the protein levels of BACE, presenilin 1 (PS1), and presenilin 2 (PS2) in the scopolamine-injected control group were higher than in the non-injected group. However, the AP10, AP20, and DO groups exhibited a decrease in the levels of BACE, PS1, and PS2 protein expression.

The receptor for advanced glycosylation end products (RAGE) is known as a multi-ligand receptor that binds to Aβ, and plasma-derived Aβ is transported by RAGE from the blood to the brain via the blood–brain barrier [58]. RAGE blocking protects neuronal cells from Aβ-induced oxidative stress [59]. The insulin degrading enzyme (IDE) is one of the extracellular proteolytic enzymes involved in Aβ removal [60]. A previous study demonstrated that low levels of IDE were observed in the brain of AD patients [61]. Therefore, increased RAGE expression or decreased IDE expression could contribute to Aβ deposition. Similar to a previous study [62], the protein level of RAGE was up-regulated by scopolamine in the control group. In contrast, the AP10, AP20, and DO groups could significantly down-regulate the levels of RAGE protein expression. In addition, extremely low expression of IDE protein, one of the principal proteases involved in the degradation of Aβ, was obtained in mice injected with scopolamine. However, the AP10- and AP20-administered groups reversed the reduction in IDE. From these results, apigenin could suppress the cognitive impairment and neuronal death induced by scopolamine via regulation of the amyloidogenic pathway and Aβ degradation protein expressions in the mouse brain.

### 2.8. Effect of Apigenin on TrkB/BDNF Pathway Protein Expressions in the Brains of Scopolamine-Injected Mice

BDNF/TrkB signaling plays a fundamental role in learning and memory abilities [63]. The BDNF, a member of the neurotrophin family, is extensively expressed throughout the brain [64]. BDNF has a regulatory function of synaptic interactions that affect learning and memory formation [65,66]. In addition, BDNF stimulates the activation of TrkB, which is associated with neuroprotection and cognitive improvement [67]. According to a previous study, scopolamine exposure inhibited BDNF and TrkB expression, leading to impairment of cognitive and memory abilities in mouse models [20].

**Figure 7:**
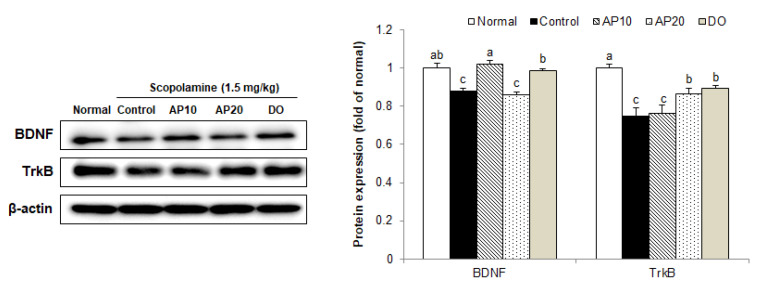
Effect of apigenin on levels of BDNF and TrkB protein expression induced by scopolamine in the brain of mice. Values are the mean ± SD. ^a–^^c^ The different letters among groups represent significant differences (*p* < 0.05) by Duncan’s multiple range test. Results of expression were calculated using β-actin as the standard. Normal: oral administration of drinking water + 0.9% NaCl i.p.; Control: oral administration of drinking water + scopolamine 1.5 mg/kg i.p.; AP10: oral administration of apigenin 10 mg/kg + scopolamine 1.5 mg/kg i.p.; AP20: oral administration of apigenin 20 mg/kg + scopolamine 1.5 mg/kg i.p.; and DO: oral administration of donepezil 5 mg/kg + scopolamine 1.5 mg/kg i.p.

In the present study, decreased levels of BDNF and TrkB protein expression were observed in a scopolamine-injected mouse brain, compared to the normal group (Figure 7). However, protein expression of BDNF was significantly enhanced in the AP10 group in comparison with the control group, which is consistent with previous reports showing the requirement for BDNF in cognitive improvement by treatment with apigenin [68]. In addition, TrkB protein expression was also significantly up-regulated in the AP20 group. These results suggest that the protective effect of apigenin against scopolamine-induced learning and memory impairment might be associated with up-regulation of BDNF/TrkB expression in the brain.

Apigenin is a bioactive flavonoid present in edible vegetables and fruits, such as parsley, celery, chamomile, guava, oranges, etc. Furthermore, apigenin has been considered a non-toxic flavonoid, even at high doses (up to 500 mg/kg) [69]. Although quantification of the dietary intake of apigenin is subject to large variations depending on age, sex, and country, the doses of apigenin used in this study were equivalent to the average daily consumption of flavonoids in humans (0.13–4.9 mg/day) [70] based on the equivalent body surface area index. Liu et al. [28] showed that treatment with apigenin (20 mg/kg/day for eight days) attenuated learning and memory impairment in Aβ_25–35_-injected mice. In addition, oral doses of apigenin at 10 and 20 mg/kg exerted neuroprotective effects by inhibiting inflammation, oxidative stress, and inflammation in rats [71]. Based on this evidence, we decided to choose apigenin at concentrations of 10 and 20 mg/kg/day, and this aspect suggests a possibility of apigenin as a therapeutic agent for the treatment of neurodegenerative diseases.

Flavonoids, including apigenin, are able to directly scavenge ROS by donating hydrogen. However, ingested flavonoids are metabolized in the intestine and liver, resulting in forms that are very different from those found in foods. There are low circulating concentrations of flavonoids in the brain, thereby it is most likely that apigenin inhibits oxidative stress through modulation of an intracellular signaling pathway such as amyloidogenic pathway or BDNF/TrkB pathway [72]. A previous study suggested that mice fed with celery-based apigenin-rich diets (aglycone form) showed improvement in the absorption of apigenin and possessed anti-inflammatory activities [73]. Although there is little known about the oral bioavailability of apigenin in humans, one of the studies demonstrated that apigenin was found in urine samples after apigenin-rich parsley intake. Another study on the bioavailability of apigenin in humans indicated that apigenin concentration was detected in the plasma following parsley consumption [74]. In addition, apigenin can cross the blood–brain barrier [75], suggesting that apigenin may have achievable bioavailable levels by oral administration and exert a neuroprotective effect as a metabolic modulator.

## 3. Materials and Methods

### 3.1. Chemicals and Reagents

**Figure 8:**
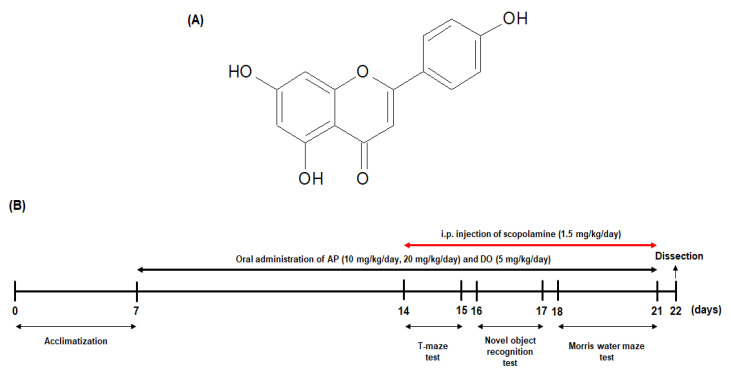
Structure of apigenin (**A**) and illustrative diagram course of behavioral tests, including the treatment of scopolamine and apigenin (**B**).

Apigenin (4′,5,7-Trihydoxyflavon, ≥95.0% purity by HPLC) (Figure 8A), scopolamine, and donepezil hydrochloride were obtained from Sigma-Aldrich Co., Ltd. (St Louis, MO, USA). Apigenin and donepezil were dissolved in water and orally administered in doses of 10 mg/kg/day, 20 mg/kg/day (apigenin), and 5 mg/kg/day (donepezil). Scopolamine was dissolved in 0.9% sodium chloride (NaCl) and administered by *i.p*. injection to mice. NaCl was purchased from LPS Solution (Seoul, Korea). Radioimmunoprecipitation assay (RIPA) buffer was purchased from Elpis Biotech (Daejeon, Korea). The polyvinylidene fluoride (PVDF) membrane was obtained from Millipore Co. (Billerica, MA, USA).

### 3.2. Animals and Experimental Protocols

Male ICR mice (5 weeks old, 25–30 g) were purchased from Orient Inc. (Seongnam, Korea). The mice were housed in plastic cages with a maintained 12 h light/dark cycle and controlled temperature (20 ± 2 °C) and humidity (50 ± 10%). The mice were free to access to water and food (5L79, Orient Inc., Seongnam, Korea). The animal protocols were confirmed by the Pusan National University Institutional Animal Care and Use Committee (PNU-IACUC; approval number: PNU-2019-2480). The mice were acclimated for a week before the experiments. They were randomly divided into five groups (*n* = 10 per group): normal, control, AP10, AP20, and DO. The mice in the normal and control groups were orally administered 100 μL of drinking water via oral gavage. The mice in the AP10 and AP20 groups were orally administered 100 μL of apigenin at doses of 10 and 20 mg/kg/day, respectively. In addition, the DO group mice (positive control group) were orally given 100 μL of donepezil at dose of a 5 mg/kg/day. Apigenin and donepezil were dissolved in the drinking water prior to oral administration, and all samples were treated daily for 14 days. The normal group mice were i.p. injected with 0.9% NaCl and the other four groups (control, AP10, AP20, and DO) were injected scopolamine (1.5 mg/kg) 30 min before every behavioral experiment, in the same way (Figure 8B). Daily water intake was observed in terms of the water remaining in the water bottle every day, and there were no significant differences in water consumption among all the groups (data not shown).

### 3.3. T-Maze Test

The T-maze test was performed according to the procedure established by Montgomery [76]. To investigate the spatial memory ability, the T-maze test was carried out. The T-shaped maze consisted of a start box, a left arm, and a right arm. On the first day, the right arm was blocked using a movable black acrylic wall, and the mice were placed at start area then left to explore the maze for 10 min. After 24 h, the black acrylic wall was removed and the mice were placed in the same apparatus, allowing them to move to the left (old route) or right (new route) arm for 10 min. The numbers entering the left and right arms were recorded.

### 3.4. Novel Object Recognition Test

The novel object recognition test was carried out using the procedure of Bevins and Besheer [77] in a black plastic square box (40 × 40 × 40 cm). On the first day, during the test, two of the same objects (A and A’) were placed at both sides of the center of the box. The mice started at the center of the objects and the numbers of touches were counted for 10 min. After 24 h, one of the objects was replaced with a new object (B), which was totally different from the others. The mice were allowed to touch both of the objects (A and B) for 10 min. The numbers of contacts with each object were recorded.

### 3.5. Morris Water Maze Test

To confirm the long-term memory, the Morris water maze test was performed [78]. The test proceeded in a circle water tank (diameter 150 cm, height 60 cm), which was filled with water and mixed with non-toxic soluble opaque black liquid gel. The water temperature was maintained at 22 ± 2 °C. The water tank was randomly divided into four quadrants and each quadrant was marked with a different sign as a space perception cue. The hidden platform was placed at approximately 1 cm below the water surface in the center of one of the quadrants. In all experiments, SMART video tracking software 3.0 (Panlab, Barcelona, Spain) was used with a camera above the circle water tank to analyze mouse movements. The program visually analyzes mouse movements to identify significant differences for each group. Training sessions were performed over three days; the intention each day was for the mice to swim and find the escape platform in 60 s using the visual clues. If the mice could not find the platform, we guided the mice carefully to the hidden platform and allow them stay there for 15 s. On the fourth day, three trials were conducted. The first trial was the same procedure as the past three days. In the second trial, we removed the hidden platform and allowed the mice to swim and find the quadrant where the platform used to be located in 60 s. In the final trial, the black water was replaced with clean water and a visible platform was provided for the mice; we recorded the time they then took to reach the platform.

### 3.6. Measurement of MDA Levels

MDA levels were measured by the method described by Ohkawa et al. [79]. After completion of the behavioral tests, dissected brain tissue was homogenized with saline solution, and mixed with 1% phosphoric acid and 0.67% thiobarbituric acid solution. After boiling at 95 °C for 45 min, the solution mixture was cooled in an ice bath, and 2 mL of 1-butanol was added followed by centrifugation at 3000 rpm for 10 min. The absorbance values of the supernatant were measured at 535 and 520 nm. The level of lipid peroxidation was calculated using an MDA standard curve.

### 3.7. Western Blot Analysis

The brain tissues were homogenized with a lysis buffer containing a protease inhibitor cocktail. The homogenates were centrifuged at 12,000 rpm for 30 min at 4 °C to obtain the supernatant. After, the total concentration of protein in the supernatant was determined with bovine serum albumin (BSA) as the standard, and samples were prepared with an equal amount of protein. Equal amounts of proteins were separated by gel electrophoresis with 10% or 13% sodium dodecyl sulfate polyacrylamide gels (SDS-PAGEs), then transferred to the PVDF membranes. The proteins were blocked by 5% skim milk for 60 min and washed with PBS. The membranes with primary antibodies were incubated over night at 4 °C (each antibody at a dilution of 1:200–1:1000; Bax, Bcl-2, cleaved caspase-3, cleaved PARP, BACE, PS1, PS2, RAGE, IDE, BDNF, TrkB, and β-actin). Then, the membranes were incubated with appropriate HRP-conjugated secondary antibodies for 60 min. Protein expressions were visualized using a chemiluminescent imaging system (Davinci Chemi, Seoul, Korea).

### 3.8. Statistical Analysis

All data in this experiment were presented as the mean ± standard deviation (SD). Statistical differences were calculated by analysis of variance (ANOVA) and Duncan’s multiple range test using the statistical program Statistical Package for the Social Sciences (version 25 SPSS Inc., Chicago, IL, USA). In the T-maze test and novel object recognition test, the perceptive ability between the training and test sessions was compared by Student’s *t*-test. *p* value of < 0.05 was considered significant.

## 4. Conclusions

In conclusion, oral administration of apigenin alleviated the cognitive and memory deficits in an AD mouse model induced by scopolamine. Apigenin also attenuated scopolamine-induced lipid peroxidation in the brains. In addition, apigenin decreased expressions of apoptosis factors, such as Bax/Bcl-2, cleaved capase-3, and cleaved PARP. Apigenin inhibited BACE, PS1, PS2, RAGE, and elevated IDE protein expression, suggesting that apigenin regulated the amyloidogenic pathway and promoted degradation of Aβ. Up-regulation of BDNF and TrkB expression by apigenin also contributed to an improvement effect on learning and memory abilities. Therefore, apigenin treatment inhibited scopolamine-induced cognitive impairment by regulating the apoptosis, amyloidogenic and BDNF/TrkB pathways. The present study suggests that apigenin could be a promising agent with the effect of improving cognitive and memory impairment in neurodegenerative diseases, such as AD.

## Figures and Tables

**Table 1:** Effects of apigenin on body and organs weight in scopolamine-injected mice.

	Normal	Control	AP10	AP20	DO
Initial body weight (g)	34.1 ± 1.4	33.3 ± 1.1	32.9 ± 0.8	33.7 ± 0.9	33.1 ± 0.7 ^NS^
Final body weight (g)	35.4 ± 2.0	33.6 ± 1.4	34.6 ± 1.5	34.1 ± 0.6	34.2 ± 0.9 ^NS^
Body weight gain (g)	1.3 ± 1.5	0.3 ± 2.0	1.7 ± 2.2	0.4 ± 1.2	1.1 ± 1.5 ^NS^
Brain (g)	0.50 ± 0.01	0.49 ± 0.02	0.51 ± 0.01	0.50 ± 0.01	0.50 ± 0.01 ^NS^
Liver (g)	2.47 ± 0.51	2.45 ± 0.21	2.25 ± 0.23	2.71 ± 0.39	2.25 ± 0.58 ^NS^
Kidney (g)	0.53 ± 0.04	0.53 ± 0.06	0.56 ± 0.07	0.59 ± 0.07	0.56 ± 0.08 ^NS^

Values are the mean ± SD. NS: non-significance. Normal: oral administration of drinking water + 0.9% NaCl i.p.; Control: oral administration of drinking water + scopolamine 1.5 mg/kg i.p.; AP10: oral administration of apigenin 10 mg/kg + scopolamine 1.5 mg/kg i.p.; AP20: oral administration of apigenin 20 mg/kg + scopolamine 1.5 mg/kg i.p.; and DO: oral administration of donepezil 5 mg/kg + scopolamine 1.5 mg/kg i.p.

## Data Availability

Data are contained within the article.

## Sample Availability

Samples of the compounds are available from the authors.
